# Asthma Control According to GINA 2023: Does Changing the Criteria Improve Asthma Control?

**DOI:** 10.3390/jcm13226646

**Published:** 2024-11-06

**Authors:** Ebymar Arismendi, Paula Ribo, Alberto García, Alfons Torrego, Irina Bobolea, Rocío Casas-Saucedo, Rosa Palomino, César Picado, Rosa Muñoz-Cano, Antonio Valero

**Affiliations:** 1 Pulmonology Department, Hospital Clínic, Universitat de Barcelona, 08036 Barcelona, Spain; earismen@clinic.cat (E.A.);; 2 Centro de Investigaciones Biomédicas en Red de Enfermedades Respiratorias (CIBERES), 28029 Madrid, Spain; 3 Respiratory Department, Hospital de la Santa Creu i Sant Pau, 08041 Barcelona, Spain; 4 Hospital General de Granollers, 08402 Barcelona, Spain; 5 Pharmalex GmbH, 50001 Zaragoza, Spain; 6 IRCE—Institut d’Investigacions Biomèdiques August Pi i Sunyer, 08036 Barcelona, Spain

**Keywords:** asthma, control, ACT, clinical characteristics, associated factors, GINA

## Abstract

**Background/Objectives**: Achieving disease control is the main goal in asthmatic patients in order to prevent future risks and exacerbations. There are several clinical guidelines that set different definitions of asthma control, and these differences may affect management and treatment in many patients. Our aim was to describe asthma control patterns according to the Global Initiative for Asthma (GINA) 2023 in patients considered to have uncontrolled asthma as per previous GINA 2010 guidelines. **Methods**: A total of 1299 patients from the COAS study were analyzed. The COAS study was a cross-sectional multicenter study conducted in routine clinical practice that included patients with uncontrolled asthma according to GINA 2010. These patients were then re-classified using the now updated GINA 2023 asthma control criteria. **Results**: After applying GINA 2023 control criteria, previously uncontrolled patients were now classified as having controlled asthma in 24.3% of cases and partially controlled asthma in 16.3% of cases. Only 59.4% maintained their previous diagnosis of uncontrolled asthma. ACT in the uncontrolled patients remained similar after re-classification, as did the percentage of active smokers, respiratory allergy, rhinitis, and lung function. **Conclusions**: Changes in clinical guideline criteria affect the definition of asthma control. When excluding pulmonary function abnormalities in GINA 2023 asthma control criteria, the percentage of controlled patients greatly increased.

## 1. Introduction

Achieving control of the disease is the main goal in the management of asthmatic patients. Several factors have been associated with poor asthma control, including age of onset, sex, body mass index (BMI), and smoking, among others [1]. Other studies have also found an increase in exacerbation and mortality rates in patients overusing short-acting beta 2-agonists [2]. The Global Initiative for Asthma (GINA) strategy [3] set the criteria to establish control and, in 2010, proposed five points to assess asthma control, one of which was pulmonary function, measured by peak expiratory flow (PEF) or forced expiratory volume in one second (FEV_1_). The GINA 2023 strategy [4] modified these criteria, proposing two different goals: (1) asthma symptom control and (2) future risk. The first goal only includes symptoms and excludes pulmonary function, which is now part of the future risk domain.

Previously, our group published the COAS study [5], a cross-sectional multicenter study that included 1299 patients with uncontrolled asthma (mean age 46.5 ± 17.3 years, 60.7% women, 25.8% obese) evaluated in routine clinical practice in specialized allergy and pulmonology centers in Spain. Its aim was to assess asthma control achieved in patients with uncontrolled asthma after appropriate recommendations and treatment optimization were implemented, following GINA 2010 guidelines. Despite this, 71.2% of patients remained not fully controlled. The study also revealed poor agreement (kappa = −0.151) when evaluating asthma control measured by the Asthma Control Test (ACT), one of the assessment tools available to evaluate asthma control by symptoms, and the GINA 2010 asthma control criteria [3]. A recent study, the REALISE survey [6], conducted in France with 1024 adult asthmatic patients, found that only 11% of asthmatic patients considered that their asthma was uncontrolled, while 48% were uncontrolled according to the GINA criteria. Frequently, patients overestimate their degree of asthma control, which can result in poor treatment adherence [6,7]. In addition, inconsistencies between the patient’s perception of their disease and their treatment can decrease adherence and therefore affect asthma control [8].

The aim of the present study was to assess and compare the level of control achieved when using GINA 2023 strategy criteria [4] compared to those of the previous GINA 2010 [3], considering that these changes in control criteria could affect the treatment and management of an important percentage of asthmatic patients. We evaluated the population of the COAS study [5].

## 2. Materials and Methods

### 2.1. Study Design

The COAS study database of uncontrolled asthmatic patients (GINA 2010) was analyzed and re-classified according to the updated GINA 2023 criteria. The characteristics of this new uncontrolled-asthma patient population were also studied. Details of the study design were previously reported in the COAS study. Functional, clinical, and epidemiological variables were collected. Informed consent was obtained from all subjects involved in the study. The study was conducted according to the guidelines of the Declaration of Helsinki and approved by the Institutional Ethics Committee of Hospital Clinic of Barcelona, 2008/4330, in 2008.

### 2.2. Study Population and Centers

Briefly, eligible patients were men and women aged 18 to 75 years with uncontrolled asthma as defined by the GINA 2010 strategy. Exclusion criteria: comorbid cardiopathy, untreated gastro-esophageal reflux, respiratory diseases other than asthma (including chronic obstructive pulmonary disease), and treatment with chronic oral corticosteroids or biologics. A total of 317 investigators from all over Spain participated in the study.

### 2.3. Endpoints of the Study

Patients’ medical history, demographic and anthropometric data were collected. Comorbidities such as rhinitis (allergic sensitization, severity, and duration), smoking habit, and lung function were assessed.

Asthma control was evaluated using the GINA 2023 criteria and the Spanish version of the validated ACT questionnaire [9]. The GINA 2023 asthma control criteria consist of four yes or no questions assessing daytime asthma symptoms, night-time awakenings due to asthma, use of reliever medication, and any activity limitation due to asthma over the past four weeks. Four negative responses define well-controlled asthma, one or two affirmative responses define partially controlled asthma, and three or four positive responses define uncontrolled asthma [4]. The ACT questionnaire is a self-administered tool for identifying poorly controlled asthma; its five questions assess activity limitation, dyspnea, nocturnal symptoms, use of rescue medication, and an overall rating of the patients’ perceived asthma control during the previous 4 weeks. Each question is scored from 1 (worst outcome) to 5 (best outcome), with the total score being the sum of all responses and ranging from 5 (not controlled) to 25 (well controlled).

### 2.4. Statistical Analysis

The procedures followed to introduce and manage study data are detailed in the COAS study [5]. In the present analysis, absolute frequencies (*n*) and relative frequencies expressed as percentages (%) were used for the descriptive treatment of qualitative variables. The mean and standard deviation were used for the analysis of quantitative variables. The analysis was performed with IBM SPSS (Statistical Package for Social Sciences) version 25.0 (SPSS, IBM Company, Armonk, NY, USA).

## 3. Results

### 3.1. Asthma Control According to GINA 2023

**Figure 1:**
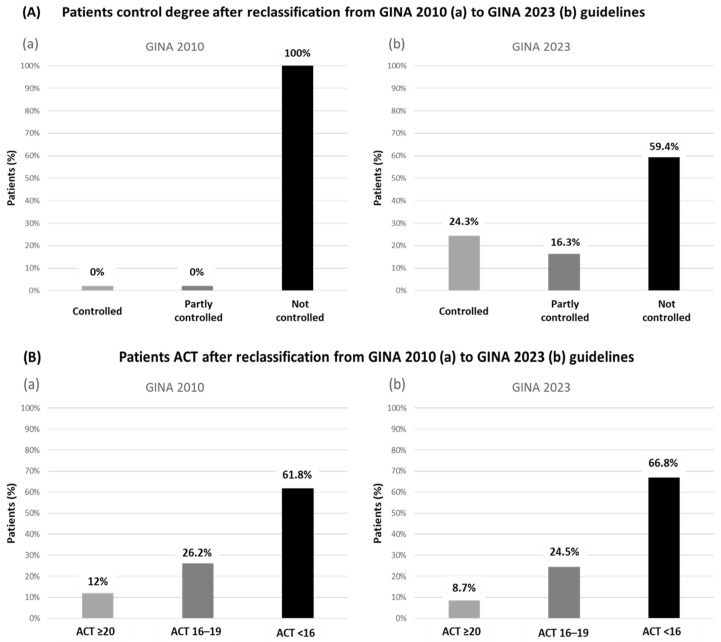
(**A**) Patients’ control degree according to GINA 2010 (**a**) and GINA 2023 (**b**) guidelines criteria. (**B**) Asthma control according to the Asthma Control Test (ACT) questionnaire and according to GINA 2010 (**a**) and GINA 2023 (**b**).

A total of 1299 patient records from the COAS study database were analyzed. These were uncontrolled asthmatic patients according to GINA 2010 criteria. After applying GINA 2023 and, hence, re-classifying patients, almost a quarter (24.3%; 316 patients) now presented well-controlled asthma, 16.3% (212 patients) presented partially controlled asthma and 59.4% (771 patients) remained as having an uncontrolled asthma diagnosis (Figure 1A).

We also analyzed, in these newly re-classified patients, asthma control measured through ACT. Notably, fewer discrepancies were found in the uncontrolled groups according to ACT and GINA 2023 (*n* = 771, 66.8%) when compared to the uncontrolled patients classified according to GINA 2010 (*n* = 1299, 61.8%) (Figure 1B).

### 3.2. Demographic, Clinical, and Asthma Control Characteristics

Patients with uncontrolled asthma according to GINA 2023 (*n* = 771, 59.4%) had a mean age of 44.8 years (SD: 16.9 years) and 59.8% were women; 15% were current smokers. Respiratory allergy was present in 55.8% of patients, while 55.6% had rhinitis, 71.8% of which was moderate and 12.1% severe. Lung function in these patients was assessed by forced vital capacity (FVC) percentage of predicted (85.9 ± 19.8), FEV_1_% (76.5 ± 20.9), and the post-bronchodilator increase in predicted FEV_1_% (14.2 ± 15.5) (Table 1).

Of the patients with uncontrolled asthma according to GINA 2010 (*n* = 1299), 60.7% were women and the mean age was 46.5 years (SD: 17.3 years), 13.8% were active smokers, 52% reported respiratory allergy, and 51.3% reported rhinitis, of which 84.3% were either moderate or severe. Lung function was equally evaluated, with patients presenting with FVC% predicted (87.2 ± 13.0), predicted FEV_1_% (76.4 ± 12.8), and post-bronchodilator increase in predicted FEV_1_% (14.9 ± 6.8) [5].

### 3.3. Risk Factors Associated with Poor Asthma Control According to GINA 2023

Possible risk factors associated with poor asthma control according to the GINA 2023 criteria may include: older age, having a diagnosis of respiratory allergy, early asthma onset, and a higher percentage of bronchodilation (Table 1). GINA 2010 considered similar risk factors: more advanced age, higher BMI, early asthma onset, greater asthma severity, worse spirometry values, and a higher percentage of bronchodilation.

## 4. Discussion

With this study, we sought to determine the impact that changes in asthma control definition could have in a cohort of patients that had been classified as having uncontrolled asthma using the GINA 2010 criteria. For that, the COAS study population was analyzed and classified according to both GINA 2010 and the new and updated GINA 2023 asthma control criteria. The main finding of our study was the notable discrepancy in the percentage of uncontrolled patients when implementing GINA 2023 criteria compared to the previous classification. Remarkably, when applying the GINA 2023 criteria, 24% of patients who were previously classified as having not controlled asthma according to GINA 2010 were now re-classified to having well-controlled asthma. Attention must be drawn to the fact that this difference of almost 25% of the patients newly classified as being well-controlled occurs because lung function was no longer included in the GINA 2023 asthma control criteria. There were no other changes in their treatment or clinical variables. Therefore, the exclusion of lung function as a control criterion in GINA 2023 has a crucial impact on asthma control achievement.

Asthma is one of the most prevalent chronic diseases in the world [10], and achieving its control is the most important goal when managing asthmatic patients. Despite this, asthma control rates remain below 50% in most developed countries [2,11,12,13,14]. We believe our study reveals the two main baseline problems regarding asthma control: asthma control definition and asthma control assessment tools.

First, the definition of control is based on arbitrary criteria and may change depending on the guideline or article selected [15,16]. The fact that GINA 2023 now considers two asthma domains, symptom control and future risk, with lung function being part of the future risk domain, represents a deviation from the previous GINA guideline´s asthma control criteria. In this regard, Alves et al. [17] observed that out of 473 patients of a severe asthma clinic, only 10% were controlled, according to GINA 2012 criteria, whereas the percentage rose to 44% when the GINA 2014 criteria were applied. These authors observed similar results to those reported herein, that is, classification using GINA 2014 resulted in a higher proportion of controlled patients, while the 2012 classification found a higher proportion of uncontrolled patients [17]. The main reason for this change was, as in our case, the exclusion of lung function as one of the control criteria. Low FEV_1_, however, may compromise asthma control despite the absence of symptoms.

Even more so, this partition of control assessment into two domains in GINA 2023 significantly differs from what was previously stated by other guidelines, such as those established by the American Thoracic Society (ATS) or the European Respiratory Society (ERS) and ATS joint task force [18,19].

Secondly, emphasis must be made on the fact that different tools used for grading adequate asthma control are limited, as was recently expressed in a recent expert consensus statement [20]. Aside from lung function, other, although subjective, tools are also available to measure asthma control, such as the ACT. Although in our previous study we found scarce agreement between the ACT and GINA 2010 criteria, this fact improved when using GINA 2023 guidelines. However, the ACT is not without limitations. Crespo-Lessman et al. [21] studied the degree of control using the ACT questionnaire in an asthmatic population and found discordance in the opinions of asthmatic patients and their physicians regarding the impact of disease on daily life activities. This disparity of opinions was associated with a poorer symptomatic control of asthma as well as with a higher future risk, especially when the physician had underestimated the impact of the disease [21].

All in all, the fact that control definition and assessment is not yet clearly established leads to different guidelines issuing different recommendations that, when not consistent, can in turn mislead the management of asthmatic patients and have a direct impact on their optimal treatment. For example, some patients with asthma could be categorized as having a mild disease, where recommendations are more controversial, thus resulting in confusion for both patients and physicians [22]. On the other end, these changes in control criteria can negatively affect patients with severe asthma, delaying the initiation of crucial new therapies, such as biologics.

It seems, then, essential to develop control criteria based on objective parameters, which may then guide changes in therapy and step-down or step-up approaches [15]. With this background in mind, the question arises: should we define asthma control solely on the presence of symptoms, hence excluding lung function, the only objective parameter we have at our disposal?

It is also important to highlight the fact that risk factors for poor asthma control have remained stable in GINA 2023, albeit minor differences. Several variables have been previously identified to affect asthma control, including age, sex, ethnicity, body mass index, smoking habit, educational level, comorbidities, treatment adherence, physical activity, and anxiety or depression, among others [23,24,25]. In the COAS study [5], older age, overweight/obesity, nonsteroidal anti-inflammatory drug (NSAID) hypersensitivity, duration of disease, poorer lung function, and a greater percentage of bronchodilation were associated with worse asthma control. This new analysis of the COAS population has found that being older, having a diagnosis of respiratory allergy, early asthma onset, or presenting a better response in the bronchodilation test were all significantly associated with a poorer disease control. NSAID hypersensitivity, however, did not seem to influence asthma control in this cohort.

The main limitation of our study is that it is a retrospective analysis of the COAS population, which influences the results and their interpretation. However, a major strength of the present study is the large number of patients analyzed with detailed clinical and functional characteristics, which allowed a deeper review and characterization of this population.

## 5. Conclusions

In conclusion, the changes in criteria of the clinical guidelines have a direct impact on the definition of asthma control and, in consequence, can cause discrepancies between treatment recommendations. The percentage of controlled patients increases significantly when using GINA 2023 compared with GINA 2010 criteria. As illustrated by our results, the exclusion of pulmonary function as a control criterion has a crucial impact in the definition of control, significantly decreasing the number of patients considered as uncontrolled. As this fact entails important repercussions when managing asthmatic patients, especially at a therapeutic level, we believe this topic deserves an in-depth analysis. In general, risk factors for poor control identified here are the same as those found in several studies. Further, longitudinal studies are necessary to ascertain whether these changes in the GINA criteria are sufficient to define asthma control. Individualized asthma control achievement remains, nonetheless, one of the major challenges in the management of asthmatic patients.

## Figures and Tables

**Table 1:** Sociodemographic and clinical characteristics of uncontrolled patients at baseline visit and classification of asthma control using GINA 2023 criteria.

Patients with Uncontrolled Asthma According to GINA 2023	Uncontrolled
Subjects *n* (%)	771 (59.4%)
Age (years) M ± SD	44.8 ± 16.9
Women *n* (%)	59.8%
Body mass index kg·m^−2^ M ± SD	26.9 ± 5.4
Smoking status *n* (%)	
Non-smokers	65.6%
Ex-smokers	19.3%
Smokers	15.0%
Diagnosis of respiratory allergy *n* (%)	55.8%
Rhinitis *n* (%)	55.6%
Years of evolution M ± SD	13.8 ± 10.6
Rhinitis severity *n* (%)	
Mild	16.1%
Moderate	71.8%
Severe	12.1%
Duration of rhinitis *n* (%)	
Intermittent	46.1%
Persistent	53.9%
Asthma, years of evolution M ± SD	13.8 ± 11.4
Asthma classification (GINA 2023)	
Mild intermittent	0.3%
Mild persistent	6.5%
Moderate persistent	83.3%
Severe persistent	9.9%
FVC % predicted M ± SD	85.9 ± 19.8
FEV_1_ % predicted M ± SD	76.5 ± 20.9
Increase post-BD FEV_1_ % predicted M ± SD	14.2 ± 15.5
ACT *n* (%)	
Controlled	8.7%
Partially controlled	24.5%
Uncontrolled	66.8%

## Data Availability

The original contributions presented in the study are included in the article, further inquiries can be directed to the corresponding author/s.
